# The Association between Using Personal Protective Equipment and Headache among Healthcare Workers in Saudi Arabia Hospitals during the COVID-19 Pandemic

**DOI:** 10.3390/nursrep11030054

**Published:** 2021-07-16

**Authors:** Nahar M. Alreshidi, Salmah Alghamdi, Faygah Shibily, Alaa Mahsoon, Nofaa Alasmee, Loujain Sharif, Ammunah Tajuddin, Najlaa Siddiq

**Affiliations:** 1 Department of Nursing, Ministry of Health, Hail 00966, Saudi Arabia; 2 Faculty of Nursing, King Abdulaziz University, P.O. Box 4105, Jeddah 22254, Saudi Arabia; saalghamdi6@kau.edu.sa (S.A.); fshibily@kau.edu.sa (F.S.); mahsoon@kau.edu.sa (A.M.); nalasmee@kau.edu.sa (N.A.); lsharif@kau.edu.sa (L.S.); 3 Ministry of Health, Jeddah 21497, Saudi Arabia; monatajuddin@hotmail.com; 4 King Faisal Specialist Hospital & Research Centre, P.O. Box 7345, AL-Madinah 42522, Saudi Arabia; naj-a-s@hotmail.com

**Keywords:** personal protective equipment (PPE), headache, COVID-19, healthcare workers, face mask, N95, coronavirus

## Abstract

It is mandatory that healthcare workers wear personal protective equipment (PPE) while caring for COVID-19 patients. Studies have shown that wearing PPE for a prolonged time may lead to symptoms of physical discomfort including headache. The aim of this study is to assess the prevalence and association between prolonged use of PPE and headaches. This was a cross-sectional study. A convenience sample of healthcare workers who worked with COVID-19 patients in clinical settings was recruited. The data were collected through an electronic survey shared as a link through social media. This study included 1060 participants, 753 (71%) female and 307 (29%) male. Participants were divided into two groups. Group A had 628 (60%) participants who did not have chronic headache before the COVID-19 pandemic, while Group B contained 432 (40%) participants who had a previous chronic headache. Headaches differed significantly between Groups A and B in frequency, type, location, and quality during the COVID-19 period. The analysis found a significant relationship between duration of PPE use and headache occurrence. The significant relationship between the duration of PPE usage and headache occurrence among healthcare workers should be considered when refining policies and procedures regarding prolonged PPE use.

## 1. Introduction

The World Health Organization (WHO) identified the outbreak of the coronavirus (COVID-19) as a global pandemic in March 2020 [1]. Worldwide, health authorities of each country mandated that healthcare workers wear personal protective equipment (PPE) while dealing with suspected or confirmed COVID-19 cases. The Saudi Ministry of Health (MOH) reinforced the importance of standard precautions using the required PPE during this time [2].

The PPE included close-fitting N95 face masks, protective eyewear (goggles, face shields, or face masks), gowns, gloves, or/and the use of powered air-purifying respirators (PAPR) [3]. However, many frontline healthcare workers found the PPE uncomfortable and cumbersome, especially when it was worn for a prolonged period of time [4].

Headache was one type of physical discomfort experienced by many frontline healthcare workers due to wearing PPE. Headache may be produced by the sustained compression of the peri-cranial soft tissues by wearing tight bands or straps around the head. The PPE face shield, for instance, is attached tightly around the head [5].

Studies have shown that wearing PPE for prolonged time periods may lead to symptoms of physical discomfort including headache [6,7,8]. However, there is a lack of literature related to PPE-associated headaches that examines the combined usage of N95 face masks and protective eyewear, specifically goggles. The aim of this study is to assess the prevalence and association between prolonged use of PPE and headaches.

The study’s objectives are as follows:

- To determine the prevalence of headaches from PPE use among healthcare workers;
- To assess the association between prolonged PPE use and headaches;
- To identify risk factors for the occurrence of headaches among healthcare workers during the COVID-19 outbreak;
- To identify symptoms associated with headache episodes.

Due to the escalation of infectious diseases and the COVID-19 outbreak, frontline healthcare workers in Saudi Arabia were mandated to wear personal protective equipment (PPE), such as close-fitting N95 face masks, disposable gloves, and protective gowns, while attending to patients, regardless of their COVID-19 status [9]. Healthcare workers have adequate knowledge of the appropriate use of PPEs and their role in safeguarding patients’ health [10]. In Saudi Arabia, nurses understand the responsibility they share with other healthcare workers to reduce infection risks by adhering to PPE requirements [10]. However, many nurses experience physiological issues, such as headache, due to long shifts and prolonged PPE use. Headaches in general are widely experienced, and nearly 90% of the world population has experienced a headache at least once in their lifetime [11,12]. Nurses who wear N95 masks are likely to experience some breathing resistance [7,13]. In turn, breathing resistance may lead to hypoventilation, a reduction in the depth and frequency of breathing. If the mask is worn continuously for over one hour, labored breathing causes the accumulation of carbon dioxide in the bloodstream [14,15]. Ultimately, a headache develops due to the increased concentration of carbon dioxide. Studies exploring this association indicate that breathing through the N95 mask impedes gaseous exchange and increases the metabolic system’s work, especially when the healthcare worker is pregnant [16]. In the Middle Eastern region, some healthcare workers view wearing the N95 mask as counterproductive as they find it difficult to tolerate.

Full body PPEs may cause heat stress and breathing difficulties that result in headaches. In addition, prolonged use of PPE worsens headaches among healthcare workers with a history of tension headaches or migraines [17,18]. Furthermore, the location of headache-associated discomfort and pain corresponds anatomically to the regions compressed by mask straps or protective goggles. The association between PPE and headache is two-fold; it results from both prolonged and incorrect PPE use. According to a recent study, headache is one of the main outcomes of the incorrect use of N95 masks [19]. This outcome, as well as the discomfort of wearing face masks and head straps, reduces nurses’ tolerability for using PPEs. Other scholars argue that prolonged use of N95 masks among nurse practitioners stems from its association with impaired mental performance and increased headache [20]. Headaches associated with PPE usage have significant and adverse impacts on healthcare workers’ health and performance outcomes. A study contended that most healthcare workers develop exacerbation of their pre-existing headache disorders [21], which affects their level of work performance. Approximately 91% of respondents in a study exploring the association between headache and PPE stated that increased protective wear usage impacted their control of existing headache issues [8]. These studies suggest that despite the protective potential of PPEs, they are associated with significant discomfort and physiological outcomes that hinder nurses’ optimal performance and productivity.

Studies exploring the effect of PPE on people’s health have focused on its association with headaches. For instance, a study of healthcare providers wearing N95 face masks during the 2003 severe acute respiratory distress syndrome (SARS) epidemic in Singapore reported a prevalence rate of 37.3% for face-mask-associated headaches [6]. Another study found that nurses who wore N95 face masks while working in a medical intensive care unit reported headache as one of their main sources of physical discomfort [22].

However, these studies are largely based on Western societies, leaving a significant literature gap. Research on the association between PPE and headache has rarely investigated the experiences of healthcare workers in other regions, such as Saudi Arabia, hence the need for this analysis.

## 2. Materials and Methods

### 2.1. Research Design

This study was a quantitative, cross-sectional survey.

### 2.2. Settings

The study was conducted in Ministry of Health hospitals, Saudi Arabia, which required the use of PPE during the COVID-19 outbreak.

### 2.3. Participants

A convenience sample of healthcare workers who worked with COVID-19 patients in clinical settings was recruited. Inclusion criteria included healthcare workers aged 21 years or more who worked in Saudi Arabia, in a hospital during the COVID-19 outbreak, wore PPE, and understood the English language.

### 2.4. Sample Size

G*Power was utilized to estimate the sample size [23]. The input parameters were an alpha of 0.05, power of 0.85, and a medium effect size of 0.3. The minimum sample size was calculated to be 75.

### 2.5. Research Instrument

An electronic self-administered questionnaire was used. The questionnaire contained six sections: (1) demographic questions, such as age, gender, and occupation; (2) medical history; (3) PPE usage pattern since the start of the COVID-19 outbreak in Saudi Arabia; (4) headache types; (5) characteristics of pre-existing headaches; (6) perceived changes in pre-existing headache characteristics since the start of the COVID-19 outbreak. The questionnaire was evaluated by experts in the healthcare field for content validity.

### 2.6. Data Collection Procedures

The data were collected through an electronic survey shared as a link through social media from September 2020 to November 2020.

### 2.7. Ethical Considerations

Ethical approval was obtained from the Ministry of Health (Log No. 2020-28). The confidentiality of participants was maintained throughout the study process.

### 2.8. Data Analysis

Data were analyzed using SPSS version 27 (2020; IBM Corp., Armonk, NY, USA). Descriptive statistics were calculated to describe the study variables. The associations between the demographic variables and headache types were examined using one-way analysis of variance (ANOVA), Pearson’s chi-square test, and Fisher’s exact test. The statistical significance level was set at *p* < 0.05. The dataset for this study is missing 10% of its data. The data were missing at random, according to analysis by SPSS. It appeared that the data missing were due to the participants’ responses. The missing data came from headache characteristics and were included in the person chi-square analysis; therefore, the result should be interpreted cautiously.

## 3. Results

This study included 1060 participants, 753 (71%) female and 307 (29%) male, as shown in Table 1. Most participants were married (63%), and the majority were between 31 and 40 years old (42%). The majority were nurses (68%). Respondents practiced in critical care units (21.5%), patient wards (18%), outpatient clinics (17%), emergency departments (13.5%), and isolation wards (10%).

This study focused on participants’ experience of headache, taking into consideration whether they had chronic headache before the COVID-19 period (Table 2). Participants were divided into two groups. Group A had 628 (60%) participants who did not have chronic headache before the COVID-19 pandemic, while Group B contained 432 (40%) participants who had previous chronic headache. A chi-squared analysis showed that the medical histories of Group A and Group B participants differed significantly, as shown in Table 2. More individuals in Group B had asthma (*p* < 0.001), ischemic heart disease (*p* < 0.001), eczema (*p* < 0.001), depression (*p* < 0.001), and anxiety (*p* < 0.001).

Groups also differed on lifestyle factors (Table 2). Significant differences were found in smoking history (*p* = 0.041), insufficient hydration (*p* < 0.001), emotional stress (*p* < 0.001), physical stress (*p* < 0.001), lack of exercise (*p* < 0.001), and irregular mealtimes (*p* < 0.001). More individuals in Group A experienced headache during the COVID-19 period than those in Group B, and Group A tended to have more individuals who smoked, experienced emotional and physical stress, and did not maintain hydration and regular meals. The exception was that more individuals who lacked exercise experienced headache during the COVID-19 period in Group B than in Group A.

In addition, participants’ responses to headache stimulating factors (sound, light, movement sensitivity, and neck discomfort) significantly differed (*p* < 0.001), with Group B demonstrating a greater response to stimulating factors than Group A.

### 3.1. Headache Character among Healthcare Providers

A significant difference between Groups A and B in the frequency of headaches, expressed as days with headache per month, was found during the COVID-19 period (*p* < 0.001; Table 3). A total of 33% of participants reported experiencing headache 1–4 days per month. A further 26% reported experiencing headache 5–9 days per month. Group B showed more individuals experiencing headache 1–4 days per month (34%) and experiencing headache 5–9 days per month (46%), while Group A showed 33% and 12%, respectively.

Headaches differed significantly between Groups A and B in type, location, and quality during the COVID-19 period (*p* < 0.001; Table 3). The most common type of headache, reported by about half of participants (52%), was a tension-type headache. The second most common headache type was migraine (23%). Furthermore, about half of participants (52%) experienced headache unilaterally; Group B (70%) showed this more than Group A (39%). Of the remaining participants, more in Group A (41%) experienced headache bilaterally. Finally, the most common headache qualities were aching (42%), throbbing (21%), and stabbing (20%). Aching and throbbing were reported more frequently by participants in Group A (48% and 20%, respectively) than Group B (35% and 23%, respectively). However, Group B (35%) participants reported experiencing stabbing headaches more often than Group A (9%).

Headache duration and intensity also differed significantly (*p* < 0.001) between Groups A and B as shown in Table 3. Almost one-third (27%) of participants experienced headache for 1–2 h. Among those participants, 39% of Group B reported experiencing headache for 1–2 h. Of the participants who experienced headache for less than an hour (23%), the majority were in Group B (24%). Regarding intensity, the majority of participants rated their headache intensity as ranging from mild (32%) to moderate (47%). Those who rated the intensity as mild were mostly in Group A (41%), while those who rated the intensity as moderate were mostly in Group B (64%).

The two groups significantly differed from each other in the number of headache episodes and the duration change during the COVID 19 period (*p* < 0.001; Table 3). The change in headache days per month was measured on a five-point Likert scale (significant decrease in frequency, slight decrease in frequency, no change, slight increase in frequency, significant increase in frequency). Participants’ responses were distributed as follows: 32% reported no change in frequency, 27.5% reported a slight increase, and 25% reported a significant increase; the other responses were less than 10% each. The majority of respondents who reported no change were in Group A with 41%. The majority of respondents who reported a slight increase in frequency or a significant increase in headache episodes were in Group B with 30% and 44%, respectively. The change in headache duration during COVID-19 was also measured on a Likert scale. The participants’ responses were as follows: 32% reported no change in duration, 28% reported a slight increase, and 25% reported a significant increase, with the other responses at 10% or less. The plurality of participants who reported no change in headache duration during COVID-19 were in Group A at 41%. The majority of respondents who reported a slight increase or a significant increase in duration were in Group B (31% and 46%, respectively).

The two groups differed significantly in terms of PPE used, namely face masks (*p* < 0.001), eye protection (*p* < 0.001), and a combination of both (*p* < 0.001; Table 4). The use of a face mask, eyewear, and a combination was measured on a four-point Likert scale (from unlikely to very likely). The majority of participants were distributed between two categories for face mask use: likely (29%) and very likely (43%). Of participants likely to use a face mask, 67% were in Group A; groups were equally represented among those very likely to use a face mask. Similarly, Groups A and B showed varied responses to the use of eye protection and a combination of face mask and eye protection. Group A participants comprised the majority of respondents on all Likert scale levels (likely, maybe, and unlikely), except one (very likely).

How often participants used PPE before and during the COVID-19 period was measured on a 5-point Likert scale (from no change in frequency to a significant change in frequency). The results are reported in Table 4. A significant difference was found in PPE use between groups since the start of the COVID-19 pandemic. More than half (56%) of participants reported a significant increase in the frequency of use, and more than half of those respondents were from Group A (52%).

Moreover, a significant difference was found between Groups A and B in their use of different types of eyewear (*p* = 0.020) and face masks (*p* = 0.047). The majority of healthcare providers used face shields (78%) and surgical face masks (65%) during their shifts. Group A (58%) participants reported a greater use of face shields than Group B (42%) and a greater use of surgical masks (60% and 40%, respectively). Furthermore, Groups A and B showed significant differences in the duration of PPE use (*p* < 0.001; Table 4). Duration was divided into four different lengths of time, and 43% of participants stated that they used PPE for 5–9 h per shift; 40% reported that they used PPE for 5–9 h (hours/day over a month).

### 3.2. Association between Prolonged PPE Use and Headache

A logistic regression analysis was used to examine the association between the duration of PPE use and headache (Table 5). The analysis found a significant relationship between the duration of PPE use and headache occurrence for the periods of 5–9 h (*p* < 0.001) and 10–12 h (*p* = 0.013). These results indicate that when healthcare workers use PPE for 5–9 h a day, they are four times more likely to experience headache than those who use PPE for shorter amounts of time.

### 3.3. Symptoms among Healthcare Providers

Participants’ symptoms during their headache episodes significantly differed between Groups A and B (Table 6), specifically nausea/vomiting (*p* < 0.001), light sensitivity (*p* = 0.006), sound sensitivity (*p* = 0.040), neck discomfort (*p* < 0.001), and movement sensitivity (*p* < 0.001). More individuals in Group B reported nausea/vomiting (53%), neck discomfort (54%), and movement sensitivity (55%) than Group A. Finally, this study tested the association between headache occurrence and symptom occurrence. Univariate logistic regression analysis was used to test each headache and symptom occurrence and found a significant association between headache occurrence and all symptoms (Table 7).

## 4. Discussion

One objective of this study was to identify headache risk factors. Participants were categorized into Group A, those who did not have chronic headaches before the COVID-19 period, and Group B, those who had prior chronic headaches. Healthcare workers who had chronic headache before COVID-19 were found to have more pre-existing conditions, including asthma, ischemic heart disease, depression, and anxiety. Since a majority of those who experienced headaches before the pandemic had pre-existing conditions, medical history was essential in the establishment of the relationship between PPE and headaches. In addition to medical history, age and marital status have been found to influence the prevalence of headaches [5]. Social issues are vital to the health of practitioners. For instance, a recent psychological study found that marriage contributed to healthcare workers’ stability [17]. Therefore, knowledge of these parameters was necessary to determine the specific influence of PPE on healthcare workers’ health.

This study found an association between prolonged use of PPE and headaches in healthcare workers. A previous review supports these findings during the COVID-19 period. Ong and colleagues have done a review and found a total of six published studies, all of which revealed an association between headache and the use of PPEs among HCWs during the COVID-19 pandemic [24]. One possible reason for the association might be that the prolonged use of N95 causes an increased concentration of Carbon monoxide in the blood, which leads to headache [13]. It is expected that during the pandemic, the frequency of healthcare workers donning full PPE would increase [6], a possible cause to study participants’ frequency and intensity of headaches increasing with longer hours of PPE use.

Additionally, participants’ type and frequency of headaches were vital in the investigation of risk factors. The frequency of headaches reported by healthcare workers during the COVID-19 period varied. The majority of participants experienced tension-type headaches, which are likely to stem from wearing PPE, rather than migraines [24]. While the majority of participants reported a difference in the occurrence of headache episodes before and during the pandemic, the frequency differed between the two groups. Participants with prior headaches in Group B reported an increase in the occurrence of headache episodes. The majority of participants in Group B reported 5–9 headaches per month, while more Group A participants experienced headaches 1–4 days per month. Thus, those with a history of headaches experienced more headaches during the COVID-19 period. Both the intensity and the magnitude of headache discomfort varied with participants’ medical history and lifestyle, supporting previous findings from Saudi Arabia [2].

The presence of a significant relationship between duration of PPE usage and headache occurrence suggests that those with pre-existing conditions will likely continue to endure increased frequency and intensity of headache. This result is supported by a previous finding [19] that disclosed that practitioners with pre-existing headache disorders were likely to suffer more as a result of prolonged use of PPE. Therefore, wearing them for longer periods increases the chances that practitioners will experience headaches. In addition, a study reported the sense of duty felt by nurses in Saudi Arabia, some of whom had pre-existing conditions that escalated their headaches [10].

These previously reported findings suggest that healthcare workers in Group B, who had a greater number of pre-existing conditions, are at a higher risk of adverse effects of PPEs relative to their Group A counterparts. Overall, the results demonstrated that those who experienced headaches before the pandemic were likely to experience headaches of greater intensity on a greater number of days per month. This knowledge can be used to devise appropriate measures to address the different levels of headache challenges that the two groups experience. The association of PPE and headaches is likely to continue or exacerbate in the wake of the virus due to the health protocols prescribed by WHO.

In addition to headaches, healthcare workers reported other discomforts due to wearing PPE such as nausea/vomiting, light sensitivity, sound sensitivity, neck discomfort, and movement sensitivity. In the current study, participants in both groups reported an increase in discomfort, with those who combined face masks and eye protection reporting the most discomfort. Similar results were reported by Williams et al. (2020), noting a range of physiological discomforts, when wearing PPEs including facemask and eye protection for a prolonged period [13]. Furthermore, according to the literature, PPE use was associated with discomfort that could affect performance [7,8,21]. Thus, there is a need to discuss the consequences of wearing PPE.

Practitioners wore PPE in specific units, before the pandemic, especially the intensive care unit (ICU). Current guidelines necessitate that those attending to patients, regardless of their status, should wear PPE, resulting in an increase in the amount of time that healthcare workers must wear protection [10]. Despite the resulting discomfort, healthcare practitioners adhere to WHO protocols in work settings, supporting the Hippocratic Oath, which prioritizes the needs of the patient. Additionally, the current findings regarding the differences in the level of use of protection between the two groups refute Tong et al.’s (2015) claim of the counterproductivity of N95 masks [13].

The symptoms manifested by participants illustrate the relationship between wearing PPE and resultant discomfort. The main symptoms of Group B participants were nausea/vomiting, neck discomfort, and movement sensitivity, while Group A participants’ main symptoms were light and sound sensitivity. Two studies reported that PPEs caused heat stress and breathing difficulties despite having powered air-purifying respirators [17,18]. Therefore, wearing them for longer periods increases the chances that practitioners will experience headaches. Moreover, the location and intensity of headache and discomfort are associated with regions compressed by the straps of masks and eyewear. Consequently, these items may be responsible for the manifestation of unilateral headaches that affected both healthcare worker groups in the current study.

The results of the current study support previous findings [19] that found that practitioners with pre-existing headache disorders were likely to suffer more as a result of prolonged use of PPE. Therefore, as nurses and other health workers fulfill their responsibilities to safeguard patients following WHO protocols, their role comes at a cost to their comfort, which may in turn affect their performance and thus public health. The situation may only worsen due to the increasing rate of the spread of infection, especially if a cure is not found and the virus continues to mutate. Our findings suggest that the consequences of the PPE protocol for healthcare workers in Saudi Arabia are similar to those in Western regions. Therefore, it is vital to determine alternative means of protection to safeguard the health of practitioners [7]. Moreover, engineers could use these findings to facilitate improvement in PPE ergonomics to reduce the occurrence of headaches. Training for HCWs with “PPEs fit-test” was also suggested.

This study has several implications for research, clinical practice, and policy. Future research can replicate this study using randomized controlled trials to assess the causal relationship between headache and PPE use. Further, future research could develop an intervention to alleviate headache occurrence with PPE use. Finally, policymakers in clinical settings can use these findings to inform PPE policy and use.

This study has some limitations. Since this was a cross-sectional design, no causal inference can be made between headache occurrence and PPE use. In addition, other factors that may contribute to headache occurrence, such as sleep disturbance, were not controlled for. The study used a self-report questionnaire, where recall bias is common. Furthermore, a PPEs usage log was not used; however, the period of COVID-19 requires using the PPE throughout the working hours. Another potential limitation is the lack of differentiation between the types of headache in the questionnaire. Although study participants were from different regions of Saudi Arabia, the results may not be generalizable as PPE policies and procedures, availability, and exposure to COVID-19 may vary between healthcare workers.

## 5. Conclusions

This study presented important findings on the prevalence and association of headache among healthcare workers using PPE during the COVID-19 pandemic, symptoms associated with headache, and risk factors for headache occurrence. The study provides several implications for future nursing research, practice, and policy. The findings suggested a significant relationship between the duration of PPE usage and headache occurrence among healthcare workers, which should be considered when refining policies and procedures regarding prolonged PPE use.

## Figures and Tables

**Table 1:** Descriptive Statistics for Participants’ Demographic Information.

Item	*n*	%
Gender		
Female	753	71
Male	307	29
Marital Status		
Divorced	14	1.3
Married	666	62.8
Single	373	35.2
Other	7	0.7
Age		
21–30	365	34.4
31–40	443	41.8
41–50	201	19.0
51–60	46	4.3
>60	5	0.5
Nationality		
Non-Saudi	646	60.9
Saudi	414	39.1
Profession		
Allied Health	59	5.6
Consultant	45	4.2
House Officer	28	2.6
Nurse	724	68.3
Resident	94	8.9
Specialist	82	7.7
Other	28	2.6
Unit		
Critical Care Unit	228	21.5
Emergency Department	143	13.5
In-Patient Ward	194	18.3
Isolation Ward	108	10.2
Out-Patient Clinic	177	16.7
Other	210	19.8
Participants’ Residence in Saudi Arabia		
Central region	344	32.5
Eastern region	168	15.8
Northern region	284	26.8
Southern region	115	10.8
Western region	149	14.1

Note. *n* = 1060.

**Table 2:** Descriptive Statistics of Study Variables and Comparison of Healthcare Providers with Headache and No Headache before the COVID-19 Pandemic using the Chi-Square Test.

Variables	Group A No Prior Headache (*n* = 628) *n* (%)	Group B Prior Headache (*n* = 432) *n* (%)	Total	*p*
**Medical History**				
Asthma				
No	604 (64.1)	338 (35.9)	942	<0.001
Yes	46 (39.3)	71 (60.7)	117	
Ischemic Heart Disease				
No	647 (61.9)	398 (38.1)	1045	<0.001
Yes	3 (20)	12 (80)	15	
Eczema				
No	637 (62.9)	376 (37.1)	1013	<0.001
Yes	13 (27.7)	34 (72.3)	47	
Stroke				
No	645 (61.6)	402 (38.4)	1047	0.089
Yes	5 (38.5)	8 (61.5)	13	
Depression				
No	637 (63.1)	372 (36.9)	1009	<0.001
Yes	13 (26.5)	36 (73.5)	49	
Anxiety				
No	620 (69.4)	273 (30.6)	893	<0.001
Yes	29 (17.8)	134 (82.2)	163	
**Lifestyle**				
Smoking History				
No	601 (62.3)	364 (37.7)	965	0.041
Yes	49 (51.6)	46 (48.4)	95	
Insufficient Hydration				
No	379 (67.3)	184 (32.7)	563	<0.001
Yes	271 (54.5)	226 (45.5)	497	
Emotional Stress				
No	304 (76.4)	94 (23.6)	398	<0.001
Yes	346 (52.3)	316 (47.7)	662	
Physical Stress				
No	267 (79.2)	70 (20.8)	337	<0.001
Yes	383 (53)	340 (47)	723	
Lack of Exercise				
No	343 (78.7)	93 (21.3)	436	<0.001
Yes	307 (49.2)	317 (50.8)	624	
Irregular Mealtimes				
No	345 (73.1)	127 (26.9)	472	<0.001
Yes	305 (51.9)	283 (48.1)	588	
**Stimulating Variables**				
Sound Sensitivity				
No	438 (64.2)	244 (35.8)	682	<0.001
Yes	136 (46.4)	157 (53.6)	293	
Light Sensitivity				
No	438 (66.8)	218 (33.2)	656	<0.001
Yes	138 (42.9)	184 (57.1)	322	
Neck Discomfort				
No	445 (71.7)	176 (28.3)	621	<0.001
Yes	131 (36.9)	224 (63.1)	355	
Movement Sensitivity				
No	484 (64.4)	267 (35.6)	751	<0.001
Yes	94 (40.7)	137 (59.3)	231	
**Medication Usage**				
Paracetamol				
No	223 (81.1)	52 (18.9)	275	<0.001
Yes	427 (54.4)	358 (45.6)	785	
NSAIDs				
No	532 (69.4)	235 (30.6)	767	<0.001
Yes	118 (40.4)	174 (59.6)	292	
ARB				
No	630 (61.5)	395 (38.5)	1025	0.606
Yes	20 (57.1)	15 (42.9)	35	
Beta blocker				
No	621 (61.9)	383 (38.1)	1004	0.181
Yes	29 (52.7)	26 (47.3)	55	

Note. *n* = 1060; statistical significance set at *p* < 0.05; ARB: Angiotensin receptor blockers, NSAID: Non-steroidal anti-inflammatory drugs.

**Table 3:** Comparison of Headache Characteristics Among Healthcare Providers using the Chi-Square Test.

Variable		Group A No Prior Headache (*n* = 628)	Group B Prior Headache (*n* = 432)	Total *n* (%)	*p*
**Headache Days/Month**					
Less than 1 day per month	N	204	47	251 (23.70)	*p* < 0.001
	%	32.48	10.89		
1–4 days per month	N	205	149	354 (33.40)	
	%	32.64	34.49		
5–9 days per month	N	76	199	275 (25.90)	
	%	12.10	46.06		
10–14 days per month	N	24	25	49 (4.60)	
	%	3.82	5.78		
More than 15 days per month	N	17	11	28 (2.60)	
	%	2.71	2.55		
Missing	N	102	1	103 (9.70)	
	%	16.24	0.23		
**Type of Headache**					
Cluster headache	N	70	23	93 (8.80)	*p* < 0.001
	%	11.15	5.32		
Migraine	N	101	144	245 (23.10)	
	%	16.08	33.33		
Migraine with aura	N	29	21	50 (4.70)	
	%	4.62	4.86		
Tension type headache	N	310	243	553 (52.20)	
	%	49.36	56.25		
Missing	N	118	1	119 (11.20)	
	%	18.79	0.23		
**Headache Location**					
One-sided (unilateral)	N	247	304	551 (52)	*p* < 0.001
	%	39.33	70.37		
Two-sided (bilateral)	N	262	126	388 (36.6)	
	%	41.72	29.17		
Missing	N	119	2	121 (11.40)	
	%	18.95	0.46		
**Headache Quality**					
Aching	N	300	153	453 (42.70)	*p* < 0.001
	%	47.77	35.42		
Burning	N	23	25	48 (4.50)	
	%	3.66	5.79		
Stabbing	N	55	153	208 (19.60)	
	%	8.76	35.41		
Throbbing	N	127	100	227 (21.40)	
	%	20.22	23.15		
Missing	N	123	1	124 (11.70)	
	%	19.59	0.23		
**Duration of Headache Episode**					
Less than 30 min	N	171	39	210 (19.80)	*p* < 0.001
	%	27.23	9.03		
30–59 min	N	136	105	241 (22.70)	
	%	21.66	24.31		
1–2 h	N	117	169	286 (27)	
	%	18.63	39.12		
More than 2 h	N	103	118	221 (20.80)	
	%	16.40	27.31		
Missing	N	101	1	102 (9.60)	
	%	16.08	0.23		
**Headache Intensity**					
Incapacitating	N	6	2	8 (0.80)	*p* < 0.001
	%	0.96	0.46		
Mild	N	256	87	343 (32.40)	
	%	40.76	20.14		
Moderate	N	222	278	500 (47.20)	
	%	35.35	64.35		
Severe	N	44	64	108 (10.20)	
	%	7.00	14.81		
Missing	N	100	1	101 (9.50)	
	%	15.92	0.23		
**Headache Episodes Change Days/Month**					
No change in frequency	N	256	85	341 (32.20)	*p* < 0.001
	%	40.76	19.68		
Slight decrease in frequency	N	75	14	89 (8.40)	
	%	11.94	3.24		
Slight increase in frequency	N	160	131	291 (27.50)	
	%	25.48	30.32		
Significant decrease in frequency	N	60	11	71 (6.70)	
	%	9.55	2.55		
Significant increase in frequency	N	77	191	268 (25.30)	
	%	12.26	44.21		
**Headache Duration Change during COVID-19 Pandemic**					
No change in frequency	N	259	84	343 (32.40)	*p* < 0.001
	%	41.24	19.44		
Slight decrease in frequency	N	91	15	106 (10)	
	%	14.49	3.47		
Slight increase in frequency	N	164	133	297 (28)	
	%	26.11	30.79		
Significant decrease in frequency	N	43	3	46 (4.30)	
	%	6.85	0.69		
Significant increase in frequency	N	71	197	268 (25.30)	
	%	11.31	45.60		

Note. The Bonferroni test was used with an adjusted α level; *p* < 0.05 is considered significant. Personal protective equipment use among healthcare providers.

**Table 4:** Comparison of Participants’ Personal Protective Equipment Usage using the Chi Square Test.

Variable		Group A No Prior Headache (*n* = 628)	Group B Prior Headache (*n* = 432)	Total *n* (%)	*p*
**Face Mask**					
Likely	N	205	101	306 (28.90)	*p* < 0.001
	%	67	33		
Maybe	N	100	71	171 (16.10)	
	%	58.50	41.50		
Unlikely	N	93	35	128 (12.10)	
	%	72.70	27.30		
Very likely	N	230	225	455 (42.90)	
	%	50.50	49.50		
**Eyewear Protection**					
Likely	N	227	100	327 (30.85)	*p* < 0.001
	%	69.40	30.60		
Maybe	N	135	70	205 (19.30)	
	%	65.90	34.10		
Unlikely	N	144	76	220 (20.80)	
	%	65.50	34.50		
Very likely	N	122	186	308 (29.10)	
	%	39.60	60.40		
**Combination of Face Mask and Eyewear**					
Likely	N	239	104	343 (32.40)	*p* < 0.001
	%	69.70	30.30		
Maybe	N	140	81	221 (20.80)	
	%	63.30	36.70		
Unlikely	N	108	65	173 (16.30)	
	%	62.40	37.60		
Very likely	N	141	182	323 (30.50)	
	%	43.70	56.30		
**PPE Usage Change since COVID-19 Pandemic**					
No change in frequency	N	125	42	167 (15.80)	*p* < 0.001
	%	74.9	25.1		
Slight decrease in frequency	N	62	16	78 (7.40)	
	%	79.5	20.5		
Slight increase in the frequency	N	102	79	181 (17.10)	
	%	56.4	43.6		
Significant decrease in frequency	N	27	11	38 (3.60)	
	%	71.1	28.9		
Significant increase in frequency	N	312	284	596 (56.20)	
	%	52.3	47.7		
**Type of PPE Protective Eyewear**					
Face shield	N	479	344	823 (77.60)	*p* = 0.020
	%	58.2	41.8		
Goggles	N	135	87	222 (20.90)	
	%	60.8	39.2		
Visor	N	14	1	15 (1.40)	
	%	93.3	6.7		
**Type of PPE Face Mask**					
None	N	5	4	9 (0.80)	*p* = 0.047
	%	55.6	44.4		
N95	N	191	144	335 (31.60)	
	%	57	43		
**Reusable fabric**	N	17	2	19 (1.80)	
	%	89.5	10.5		
Surgical	N	415	282	697 (65.80)	
	%	59.5	40.5		
**Type of N95 Mask**					
3M Aura NOISH 1870 + Aura (white mask)	N	76	36	112 (10.60)	*p* = 0.076
	%	67.9	32.1		
3M NIOSH 1860S (green mask)	N	294	196	490 (46.20)	
	%	60	40		
Not using N95	N	258	200	458 (43.20)	
	%	56.3	43.7		
**PPE Wear Hours/Day**					
30 min to 4 h	N	201	86	287 (27.10)	*p* < 0.001
	%	70	30		
5–9 h	N	207	249	456 (43)	
	%	45.4	54.6		
10–12 h	N	131	72	203 (19.20)	
	%	64.5	35.5		
>12 h	N	89	25	114 (10.80)	
	%	78.1	21.9		
**PPE Wear Hours/Day over Month**					
30 min to 4 h	N	199	84	283 (26.70)	*p* < 0.001
	%	70.3	29.7		
5–9 h	N	175	244	419 (39.50)	
	%	41.8	58.2		
10–12 h	N	133	57	190 (17.90)	
	%	70	30		
>12 h	N	121	47	168 (15.80)	
	%	72	28		

Note. PPE: Personal Protective Equipment. The Bonferroni test was used with an adjusted α level; *p* < 0.05 is considered significant.

**Table 5:** Logistic Regression of PPE Use and Headache.

PPE Usage Hours/Day	B	SE	Wald	df	*p*	OR	95% CI of OR	
							Lower	Upper
½ h–4 h	0.421	0.26	2.61	1	0.106	1.523	0.914	2.538
5–9 h	1.454	0.245	35.211	1	<0.001 *	4.282	2.649	6.923
10–12 h	0.671	0.27	6.192	1	0.013 *	1.957	1.153	3.32
Constant	−1.27	0.226	31.468	1	<0.001	0.281		

Note. CI: Confidence interval; df: Degree of freedom; OR: Odds Ratio; PPE: Personal Protective Equipment. SE: Standard Error; *: *p* < 0.05 is considered significant.

**Table 6:** Symptom Differences Among Healthcare Providers Who Experienced Headache using Chi Squared Test.

Symptoms	Group A No Prior Headache (*n* = 628) *n* (%)	Group B Prior Headache (*n* = 432) *n* (%)	Total	*p*
Nausea/vomiting				*p* < 0.001
No	453 (59.20)	312 (40.80)	765	
Yes	103 (46.60)	118 (53.40)	221	
Light Sensitivity				*p* = 0.006
No	391 (59.60)	265 (40.40)	656	
Yes	162 (50.30)	160 (49.70)	322	
Sound Sensitivity				*p* = 0.040
No	400 (58.70)	282 (41.30)	682	
Yes	151 (51.50)	142 (48.50)	293	
Neck Discomfort				*p* < 0.001
No	387 (62.30)	234 (37.70)	621	
Yes	165 (46.50)	190 (53.50)	355	
Movement Sensitivity				*p* < 0.001
No	448 (59.70)	303 (40.30)	751	
Yes	105 (45.50)	126 (54.50)	231	

Note. The Bonferroni test was used with an adjusted α level; *p* < 0.05 is considered significant.

**Table 7:** Logistic Regression Analysis of Symptoms Associated with Headache Among Healthcare Providers.

Symptoms	B	SE	Wald	df	*p*	OR	95% CI for OR	
							Lower	Upper
Nausea/Vomiting	0.509	0.154	10.973	1	0.001	1.663	1.231	2.248
Constant	−1.481	0.109	184.105	1	0	0.227		
Light Sensitivity	0.377	0.137	7.561	1	0.006	1.457	1.114	1.906
Constant	−0.881	0.093	88.926	1	0	0.414		
Sound Sensitivity	0.288	0.14	4.211	1	0.04	1.334	1.013	1.756
Constant	−0.974	0.096	104.032	1	<0.001	0.378		
Neck Discomfort	0.644	0.135	22.824	1	<0.001	1.904	1.462	2.48
Constant	−0.852	0.093	84.067	1	<0.001	0.426		
Movement Sensitivity	0.573	0.152	14.299	1	<0.001	1.774	1.318	2.388
Constant	−1.451	0.108	179.051	1	<0.001	0.234		

Note. CI: Confidence interval; df: Degree of freedom; OR: Odds Ratio; SE: Standard Error; *p* < 0.05 is considered significant. Headache is the independent factor.

## Data Availability

The data is available from the authors on request.
